# *Δ133p53* is an independent prognostic marker in *p53* mutant advanced serous ovarian cancer

**DOI:** 10.1038/bjc.2011.433

**Published:** 2011-10-18

**Authors:** G Hofstetter, A Berger, E Schuster, A Wolf, G Hager, I Vergote, I Cadron, J Sehouli, E I Braicu, S Mahner, P Speiser, C Marth, A G Zeimet, H Ulmer, R Zeillinger, N Concin

**Affiliations:** 1 Department of Gynecology and Obstetrics, Innsbruck Medical University, Anichstrasse 35, 6020 Innsbruck, Austria; 2 Department of Obstetrics and Gynecology, Molecular Oncology Group, Medical University of Vienna, Waehringer Guertel 18-20, 5Q, 1090 Vienna, Austria; 3 Division of Gynaecological Oncology, Department of Obstetrics and Gynaecology, Universitaire Ziekenhuzen Leuven, Katholieke Universiteit Leuven, Herestraat 49, 3000 Leuven, Belgium; 4 Department of Gynecology, European Competence Center for Ovarian Cancer, Campus Virchow Klinikum, Charité – Universitätsmedizin Berlin, Augustenburger Platz 1, 13353 Berlin, Germany; 5 Department of Gynecology and Gynecologic Oncology, University Medical Center Hamburg-Eppendorf, Martinistrasse 52, 20246 Hamburg, Germany; 6 Department of Medical Statistics, Informatics, and Health Economics, Innsbruck Medical University, Anichstrasse 35, 6020 Innsbruck, Austria

**Keywords:** ovarian cancer, delta133p53, p53 isoforms, p53, p73

## Abstract

**Background:**

We aimed to evaluate the clinical relevance of p53 and p73 isoforms that modulate the function of p53.

**Methods:**

This prospective multicentre study included 154 patients with stage III and IV serous ovarian cancer. A functional yeast-based assay and subsequent sequencing were performed to analyse the *p53* mutational status. Expression of *p53* and *p73* isoforms was determined using RT–qPCR.

**Results:**

*Δ133p53* expression constituted an independent prognostic marker for recurrence-free (hazard ratio=0.571, *P*=0.016, 95% CI: 0.362–0.899) and overall survival (hazard ratio=0.365, *P*=0.004, 95% CI: 0.182–0.731) in patients with *p53* mutant ovarian cancer (*n*=121). High *Δ40p53* expression was associated with favourable tumour grading (*P*=0.037) and improved recurrence-free survival (33.4 *vs* 19.6 months, *P*=0.029), but not overall survival (43.1 *vs* 33.6 months, *P*=0.139), in patients with *p53* wild-type cancer (*n*=33). Neither the *p53* mutational status nor *p73* isoform expression possessed prognostic significance in the examined ovarian cancer cases.

**Conclusion:**

*Δ133p53* expression was associated with prognosis in the vast majority of ovarian cancer cases, that is, patients with *p53* mutant advanced serous carcinomas. Thus, our findings underline the importance of considering the complex p53 regulatory network.

Recently, the N-terminally truncated p53 isoforms *Δ*40p53 and *Δ*133p53 were added to the complex network that modulates the function of the tumour suppressor p53. *Δ*40p53 is generated by alternative splicing of intron 2 or altered initiation of translation in exon 4 (Courtois *et al*, 2002; Yin *et al*, 2002; Ghosh *et al*, 2004), whereas *Δ*133p53 is derived from an alternative promoter located in intron 4 (Bourdon *et al*, 2005). *Δ*40p53 lacks the first 40 amino acids, but retains the second transactivation domain (Murray-Zmijewski *et al*, 2006). By contrast, *Δ*133p53 is devoid of both transactivation domains and part of the DNA-binding domain (Figure 1). Both p53 isoforms contain the C-terminal tetramerisation domain, allowing their incorporation into p53 tetrameres. The exact function of *Δ*40p53 and *Δ*133p53 has not yet been fully characterised. The present consensus is that their ratio to FLp53 determines functional outcome (Jänicke *et al*, 2009). *Low Δ*40p53 and *Δ*133p53 levels have been reported to act as potent dominant-negative inhibitors of FLp53, suppressing transcription of genes under the control of a p53-binding element as well as p53-induced apoptosis (Yin *et al*, 2002; Bourdon *et al*, 2005; Graupner *et al*, 2009). Contrarily, *high Δ*40p53 and *Δ*133p53 levels have resulted in enhanced transcription of FLp53 target genes (Chen *et al*, 2005; Ohki *et al*, 2007; Powell *et al*, 2008). Studies analysing the expression patterns of N-terminally truncated p53 isoforms in distinct types of carcinomas are rare and have not shown a clinical relevance of p53 isoforms (Boldrup *et al*, 2007; Avery-Kiejda *et al*, 2008; Ebrahimi *et al*, 2008; Chen *et al*, 2009; Song *et al*, 2009).

Similarly, the p53 family member p73 gives rise to multiple isoforms. Alternative splicing of the P1 promoter transcript generates both the full-length *TAp73* as well as *ΔN7prime;p73*, whereas an alternative P2 promoter in intron 3 produces *ΔNp73*. Importantly, *ΔN′p73* and *ΔNp73* transcripts encode the same protein product lacking the transactivation domain. This N-terminally truncated p73 protein ΔNp73 acts as a powerful dominant-negative inhibitor of both wild-type p53 and TAp73, either by direct competition for DNA binding sites or by the formation of heterocomplexes (Bailey *et al*, 2011). *Δ*Np73 has been an independent prognostic marker in distinct types of carcinomas (Uramoto *et al*, 2004; Müller *et al*, 2005; Liu *et al*, 2006; Vilgelm *et al*, 2010). In ovarian cancer, we previously reported that expression of N-terminally truncated p73 isoforms has a role in response to platinum-based chemotherapy and constitutes an independent prognostic marker in patients with *p53* mutant ovarian cancer (Concin *et al*, 2005).

Most existing studies in ovarian cancer comprise patient groups heterogeneous for well-known prognostic factors such as FIGO stage and histological subtypes. While 5-year survival rates can be as high as 80–95% among patients with early-stage disease (stage I or II), patients with advanced carcinomas (stage III or IV) have survival rates of 10–30% (Cannistra, 2004). Recently, the histological subtype was reported to be an independent prognostic marker in patients with stage III ovarian cancer. Mucinous and clear cell carcinomas possessed an impaired prognosis as compared with serous ovarian carcinomas, whereas endometrioid carcinomas showed favourable prognosis (Winter *et al*, 2007). In addition, histological subtypes display different biomarker expression profiles, thus further supporting the hypothesis that histological subtypes represent different disease entities. For instance, serous carcinomas show WT1, Mesothelin, oestrogen receptor and CA125 expression in >75%, while the mucinous subtype displays frequent expression of Matriptase, and endometrioid carcinomas express high rates of estrogen and progesterone receptor and CA125 (Köbel *et al*, 2008). Heterogeneous patient groups might substantially confound results of biomarker studies and constitute an important reason for the difficulty experienced in confirming potential prognostic markers in subsequent studies.

Thus, a homogeneous cohort of patients with primary advanced serous ovarian cancer was prospectively recruited within the multicentre study OVCAD (OVarian CAncer Diagnosis of a silent killer). We aimed to evaluate the clinical relevance of *p53* (*Δ133p53*, *Δ40p53*, and *FLp53*) and *p73* isoforms (*TAp73* and *ΔTAp73*) that modulate the function of p53 in patients with advanced serous ovarian cancer.

## Patients and methods

### Patients and tissue samples

Between August 2005 and December 2008, 154 consecutive patients diagnosed with primary advanced serous ovarian cancer at the Departments of Gynecology and Obstetrics in Berlin (*n*=55), Leuven (*n*=50), Hamburg (*n*=26), Vienna (*n*=19), and Innsbruck (*n*=4) were enrolled in the OVCAD project, a Sixth Framework Program Project of the European Union (http://www.ovcad.eu). The Ethics Committees of the respective centres approved the study protocol. All patients signed written informed consent before enrollment.

### RNA isolation and real-time RT–PCR

Tissue samples were obtained at the time of diagnosis and immediately stored in liquid nitrogen. RNA isolation was done by 1600 nucleic acid prepstation (Applied Biosystems, Foster City, CA, USA) according to the manufacturer's instructions including a DNase digestion step. Eluted RNA was precipitated with ethanol and resuspended in RNase-free water. Reverse transcription was performed as described previously (Reimer *et al*, 2007).

Primer pairs and probes for *p53* (Δ*40p53,* Δ*133p53*, and *FLp53*) and *p73* isoforms (*TAp73* and Δ*TAp73*, which recognises both Δ*Np73* and Δ*N’p73* transcripts) as well as the internal control *TBP* (TATA box-binding protein) were designed using Primer Express software (Applied Biosystems; Supplementary Table 1). Real-time TaqMan RT–PCR was performed using the ABI Prism 7900 Detection System (Applied Biosystems) according to the manufacturer's recommended protocol. Each reaction was performed in duplicate. To determine absolute copy numbers for all *p53* and *p73* isoforms, standard curves were generated as reported previously (Concin *et al*, 2004).

### Analysis of *p53* mutational status

To detect alterations in the *p53* gene resulting in a functionally inactive protein, a functional yeast-based assay and subsequent sequencing were performed as described in detail previously (Concin *et al*, 2004). Throughout the manuscript the term ‘mutant p53’ is used synonymously for ‘functionally inactivated p53’ as determined by the yeast-based assay.

### Statistical analysis

The Shapiro–Wilk test was performed to assess the normality assumption. As the distribution of *p53* and *p73* isoforms was non-Gaussian, the Mann–Whitney *U*-test was performed to compare isoform expression and age with *p53* mutational status. The *χ*^2^-test was performed to examine the relationship between the *p53* mutational status and categorical clinicopathological parameters. For correlations among isoforms, Spearman's correlation coefficients were calculated.

Cases were divided by the 50th percentile of *p53* and *p73* isoform expression levels into two approximately same-sized groups (i.e., a high-expressing group and a low-expressing group of isoform expression). In addition, a *TAp73/ΔTAp73* quotient was calculated and again divided by the 50th percentile. The *χ*^2^-test was used to examine the relationships between isoform expression and clinicopathological parameters.

Survival probabilities were calculated with the product limit method of Kaplan and Meier. The Cox proportional hazards model was used for multivariate analysis to assess the independence of different prognostic factors. Statistical Package for the Social Sciences for Windows 18.0 software (SPSS, Inc., Chicago, IL, USA) was used for all analyses. *P*-values <0.05 were considered statistically significant.

## Results

### Patient characteristics

In all, 154 patients diagnosed with serous ovarian cancer were enrolled. Median age was 57 years (range: 26–83). Of the patients, 81.8% (126 of 154) presented with FIGO stage III and 18.2% (28 of 154) with stage IV disease. In all, 3.9% (6 of 154) had well, 23.4% (36 of 154) moderately, and 72.7% (112 of 154) poorly differentiated carcinomas. Primary debulking surgery and subsequent platinum-based chemotherapy were performed in 87.7% (135 of 154) of the patients. In 12.3% (19 of 154) of the patients, neoadjuvant platinum-based chemotherapy was administered after diagnostic laparoscopy and interval surgery was performed. Complete macroscopic resection was achieved in 71.4% (110 of 154) of the patients. Residual disease was ⩽1 cm in 18.2% (28 of 154) and >1 cm in 10.4% (16 of 154) of the patients. Table 1 provides the prognostic relevance of clinicopathological variables in univariate and multivariate analysis.

Median follow-up was 24.5 months (range: 1–49). Of the patients, 24.8% (38 of 153) suffered treatment failure, defined as primary progression or early recurrence within 6 months after termination of primary platinum-based chemotherapy. In one patient, treatment response could not be determined due to short follow-up.

### Clinical relevance of *p53* mutational status

Of the ovarian cancer specimens, 78.6% (121 of 154) harboured *p53* mutations and 21.4% (33 of 154) were wild-type *p53*. *p53* mutant carcinomas were significantly associated with older age at diagnosis (median 58 *vs* 50 years, *P*<0.001) and adverse tumour grade (III *vs* I/II, *P*<0.001) compared with *p53* wild-type carcinomas. Tumour stage, residual disease, and treatment response did not differ with respect to *p53* mutational status (Table 2).

Patients with *p53* mutant ovarian cancer showed impaired recurrence-free (mean 22.4 *vs* 25.7 months) and overall survival (mean 37.1 *vs* 39.0 months) as compared with patients with *p53* wild-type cancer. Neither difference was statistically significant (*P*=0.223, *P*=0.291).

*p53* and *p73* isoform expression levels did not differ between *p53* mutant and *p53* wild-type ovarian cancer specimens. Median copy numbers stratified by *p53* mutational status are provided in Supplementary Table 2.

### *p53* isoforms influence prognosis depending on *p53* mutational status

**Figure 2:**
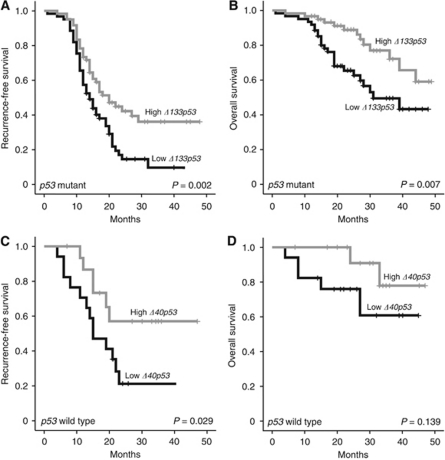
Kaplan–Meier survival graphs for *p53* isoforms. High *Δ133p53* expression was associated with improved recurrence-free (**A**) and overall survival (**B**) in 121 patients with p53 mutant ovarian cancer in univariate analyses. In patients with *p53* wild-type cancer (*n*=33), high *Δ40p53* expression levels predicted improved recurrence-free (**C**), but not overall survival (**D**) in univariate survival analyses. Cases were divided at the 50th percentile of *p53* isoform expression levels into a high- and a low-expressing group. *P*-value was determined with the log-rank test.

In patients with *p53* mutant ovarian cancer, *high Δ133p53* expression was associated with significantly improved recurrence-free and overall survival (Table 3; Figure 2A and B). A mean recurrence-free survival of 27.0 (95% CI: 22.5–31.5) months was observed in patients with *high Δ133p53* expression as compared with 17.5 (95% CI: 14.6–20.4) months in patients with *low Δ133p53* expression (*P*=0.002). Mean overall survival was 41.1 (95% CI: 37.4–44.7) months in patients with *high Δ133p53* expression and 32.7 (95% CI: 28.4–37.1) months in patients with *low Δ133p53* expression (*P*=0.007). The median survival times were not determined as <50% of patients were dead at the time of analysis, thus means are provided.

A multivariate model comprising *Δ133p53* expression and all clinicopathological parameters (age at diagnosis, tumour stage, tumour grade, and residual disease) was generated in patients with *p53* mutant cancer (Table 4). *Δ133p53* expression constituted an independent prognostic marker for recurrence-free (hazard ratio=0.571, *P*=0.016, 95% CI: 0.362–0.899) and overall survival (hazard ratio=0.365, *P*=0.004, 95% CI: 0.182–0.731). Moreover, tumour stage and residual disease were independently associated with recurrence-free survival (hazard ratio=2.287, *P*=0.001, 95% CI: 1.373–3.809 and hazard ratio=1.729, *P*=0.021, 95% CI: 1.085–2.753, respectively), and age at primary diagnosis with overall survival (hazard ratio=1.046, *P*=0.006, 95% CI: 1.013–1.079).

*Δ133p53* expression correlated with response to primary treatment in patients with *p53* mutant cancer. While only 18.3% (11 of 60) of the patients with *high* *Δ133p53* expression showed treatment failure, 34.4% (21 of 61) of the patients with *low* *Δ133p53* expression failed to respond to primary treatment. This association did not reach statistical significance (*P*=0.063).

In patients with *p53* wild-type cancer, *high* *Δ40p53* expression was significantly associated with improved recurrence-free (33.4 *vs* 19.6 months, *P*=0.029), but not with overall survival (43.1 *vs* 33.6 months, *P*=0.139; Table 3; Figure 2C and D). Due to small case numbers, multivariate analysis could not be performed.

In the entire group of serous ovarian cancer cases, *p53* isoforms did not possess prognostic relevance. Detailed survival data are given in Supplementary Table 3.

### Correlations between *p53* isoforms and clinicopathological parameters

In patients with *p53* mutant cancer, *p53* isoform expression was not associated with clinicopathological parameters. In patients with *p53* wild-type cancer, *Δ40p53* expression levels correlated with favourable tumour grading (grade I/II *vs* III, *P*=0.037). In the entire group of advanced serous ovarian cancer cases, no correlation was seen between *p53* isoforms and clinicopathological parameters.

Significant correlations between all *p53* isoforms were observed in *p53* mutant cases as well as the entire group of ovarian carcinomas, whereas the only correlation found in *p53* wild-type cases was between *Δ40p53* and *FLp53* expression. Detailed information is provided in Supplementary Table 4.

### *p73* isoforms lack prognostic significance

Neither *TAp73* nor *ΔTAp73* expression was associated with prognosis in the examined ovarian cancer cases. Given the fact that *Δ*TAp73 acts in a dominant-negative manner on TAp73, we tested the hypothesis that patients might have different clinical outcomes depending on the *TAp73*/*ΔTAp73* ratio. This ratio also did not possess prognostic relevance in the patients with advanced serous ovarian cancer. In patients with *p53* wild-type ovarian cancer, *TAp73*/*ΔTAp73* ratio correlated with favourable tumour grading (grade I/II *vs* III, *P*=0.011).

Correlations between *p73* isoforms are provided in Supplementary Table 2.

## Discussion

We herein provide first evidence for a potential clinical role of N-terminally truncated p53 isoforms with respect to the *p53* mutational status in ovarian cancer. Only one previous study has analysed the expression of p53 isoforms in ovarian cancer (Marabese *et al*, 2007). Marbese *et al* (2007) found that *FLp53* was significantly elevated in early as compared with advanced disease. However, *p53* isoforms lacked prognostic significance in their group of patients with ovarian carcinomas comprising various histological subtypes.

In the present study, *Δ133p53* expression constituted an independent prognostic marker in patients with *p53* mutant advanced serous ovarian cancer, which represents the vast majority of ovarian cancer cases. *High Δ133p53* expression resulted in a 43% risk reduction for recurrence and a 64% risk reduction for death as compared with *low* *Δ133p53* expression. To date, only scarce information is available on the function of this N-terminally truncated p53 isoform (Jänicke *et al*, 2009). Existing *in-vitro* studies evaluated the function of *Δ*133p53 either alone or in the presence of wild-type p53 (Bourdon *et al*, 2005; Chen *et al*, 2005). *Δ*133p53 has been shown to form heterocomplexes with wild-type FLp53, resulting in the dominant-negative inhibition of FLp53 (Bourdon *et al*, 2005). The present clinical study, however, demonstrates a favourable role of *Δ*133p53 in p53 mutant carcinomas. It is unclear how *Δ*133p53 might exert a beneficial function in the presence of mutant p53. We hypothesise that *Δ*133p53 may interact with mutant p53, thereby abolishing negative effects of mutant p53. Mutant p53 has been reported to counteract wild-type FLp53 and TAp73 in a dominant-negative manner, and specific mutants possess oncogenic gain-of-function mutations (Oren and Rotter, 2010). Functional studies analysing the role of *Δ*133p53 in the presence of mutant p53 are highly warranted.

A possible explanation for differing *Δ133p53* levels in cancer specimens observed in the present study might be provided by the presence of several polymorphisms within the internal promoter region located in the intron 4 of the *p53* gene, which gives rise to *Δ133p53* (Bellini *et al*, 2010). Bellini *et al* (2010) reported that these polymorphisms are associated with differences in promoter activity by changing the affinity for distinct transcription factors.

In the present study, *Δ40p53* influenced recurrence-free but not overall survival in *p53* wild-type ovarian cancer. We assume that the latter is related to the short follow-up period in the present study. Existing *in-vitro* studies suggest that *Δ*40p53 might enhance the function of wild-type FLp53 (Jänicke *et al*, 2009). *Δ*40p53 and FLp53 have been reported to readily form heterocomplexes (Courtois *et al*, 2002; Ghosh *et al*, 2004). As *Δ*40p53 lacks the MDM2-binding site, these heterocomplexes escape MDM2-mediated degradation and therefore accumulate (Yin *et al*, 2002). In addition, *Δ*40p53 supports a conformation of FLp53 that is associated with a more active state (Powell *et al*, 2008). Furthermore, Powell *et al* (2008) reported that *Δ*40p53 alters the post-translational modification profile of FLp53. Post-translational modifications at the N-terminus of FLp53, for instance, might increase the recruitment of transcriptional co-activators such as p300 and PCAF, and thus be responsible for increased promoter-binding capacity of the heterocomplexes. Furthermore, *Δ*40p53 alone has been reported to induce apoptosis through the transcriptional activation of many apoptosis-related genes that are not induced by FLp53, such as TP53BP2 (tumour protein p53 binding protein 2) and TIAL1 (TIA1 cytotoxic granule-associated RNA binding protein-like 1) (Ohki *et al*, 2007). However, the small number of *p53* wild-type cases has to be considered when interpreting our finding.

*p53* mutational status, determined with a highly sensitive yeast-based assay, did not impact prognosis in the examined patients with advanced serous ovarian cancer. In our previous retrospective study including a heterogeneous group of various histological subtypes and stages, *p53* mutational status constituted a significant prognostic marker in univariate, but not in multivariate analyses (Concin *et al*, 2005). A recent meta-analysis evaluated the role of p53 mutational status in ovarian cancer (de Graeff *et al*, 2009). In studies restricted to serous carcinomas, a modest effect of p53 on overall survival was present (HR=1.61, 95% CI: 1.09–2.38). The present study agrees with the meta-analysis restricted to advanced disease, in that it found no prognostic value of p53.

Existing studies in ovarian cancer have reported inconsistent results on the clinical relevance of p73 isoforms (Buhlmann and Pützer, 2008). High levels of N-terminally truncated *p73* isoforms were found to be independently associated with impaired prognosis in *p53* mutant cases (Concin *et al*, 2005). In contrast, high *ΔNp73* expression correlated with better overall survival irrespective of *p53* mutational status in another study (Marabese *et al*, 2007). However, the present prospective study was not able to verify a clinical role of *p73* isoforms in ovarian cancer.

Despite the myriad existing studies investigating the extremely complex network regulating p53 function, many details remain to be explored. The generation of multiple p53 isoforms adds an additional layer of complexity to this fascinating protein. It is increasingly being recognised that the classification of carcinomas into ‘p53 wild-type’ and ‘p53 mutant’ is an oversimplification that does not acknowledge the actual activity of the p53 pathway. Our clinical findings suggest that neglecting interactions between p53 and its N-terminally truncated isoforms might constitute an important reason for the difficulties encountered when attempting to correlate p53 with prognosis and treatment response. In addition, these complex interactions also have to be considered in therapeutic efforts aiming to directly affect p53 function, such as p53-gene therapy, vaccinating against p53, and the use of MDM2 inhibitors to activate the p53 antitumour response and cytoprotective function (Cheok *et al*, 2011). In future, upregulation of *Δ*133p53 in cancer cells could be an elegant means of improving outcome in patients with p53 mutant ovarian cancer. Thus, the tumour suppressor p53 remains a highly dynamic and rapidly expanding area of research.

Our findings underline the importance of considering the complex p53 regulatory network in clinical studies. In *p53* mutant advanced serous ovarian cancers, which represent the vast majority of patients, *Δ133p53* expression levels discriminate between two groups of patients with substantially different clinical outcome. *High Δ133p53* expression resulted in a 43% risk reduction for recurrence and a 64% risk reduction for death as compared with *low* *Δ133p53* expression.

## Figures and Tables

**Figure 1:**
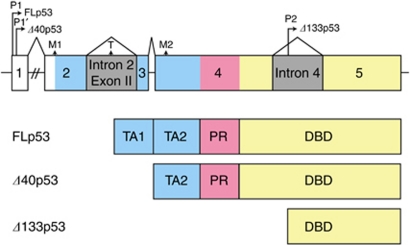
Gene architecture of the N-terminus of the *p53* gene, indicating the mode of generation for the various *p53* isoforms as well as their resulting protein structure (TA, transactivation domain (blue); PR, proline-rich domain (red); DBD, DNA-binding domain (yellow); untranscribed regions (white); introns (grey)).

**Table 1:** Prognostic relevance of clinicopathological variables in univariate and multivariate survival analyses in 154 patients with advanced serous ovarian cancer

	**Recurrence-free survival**			**Overall survival**		
	**Univariate**	**Multivariate**		**Univariate**	**Multivariate**	
	***P*-value**	**HR (95% CI)**	***P*-value**	***P*-value**	**HR (95% CI)**	***P*-value**
*Age (years)*						
⩽57 *vs* >57^a^	0.309	1.005 (0.987–1.023)	0.603	0.042	1.022 (0.995–1.050)	0.042
						
*Tumour stage*						
III *vs* IV	<0.001	2.378 (1.488–3.799)	<0.001	0.014	2.048 (1.031–4.066)	0.014
						
*Tumour grade*						
I/II *vs* III	0.017	1.558 (0.963–2.520)	0.071	0.046	1.751 (0.801–3.828)	0.046
						
*Residual disease*						
0 *vs* >0	0.001	1.779 (1.175–2.692)	0.006	0.118	1.614 (0.870–2.994)	0.118

Abbreviations: CI=confidence interval; HR=hazard ratio.

a Median age at diagnosis was 57 years.

**Table 2:** Associations between *p53* mutational status and clinicopathological parameters

		**p53 status**		
**Clinical parameters**	***n***	**Wild-type (*n*=33)**	**Mutant (*n*=121)**	***P*-value**
*Age (years)*				
Median (range)	154	50 (26–73)	58 (35–83)	<0.001
				
*Tumour stage*				
III	126	30	96	0.201
IV	28	3	25	
				
*Tumour grade*				
I/II	42	18	24	<0.001
III	112	15	97	
				
*Residual disease*				
0	110	26	84	0.285
⩽1 cm	28	6	22	
>1 cm	16	1	15	
				
*Treatment response* a				
Yes	115	26	89	0.492
No	38	6	32	

a Treatment failure was defined as primary progression or early recurrence within 6 months after termination of primary platinum-based chemotherapy. In one patient treatment response could not be determined due to short follow-up.

**Table 3:** Prognostic relevance of *Δ133p53* and *Δ40p53* expression in p53 mutant and p53 wild-type ovarian cancer cases, respectively – univariate analyses

	**Recurrence-free survival (RFS)**		**Overall survival (OS)**	
**Variable**	**Mean RFS in months (95% CI)**	***P*-value**	**Mean OS in months (95% CI)**	***P*-value**
*Δ133p53* *in p53 mutant cases (n=121)*				
<50th percentile	17.5 (14.6–20.4)		32.7 (28.4–37.1)	
>50th percentile	27.0 (22.5–31.5)	0.002	41.1 (37.4–44.7)	0.007
				
*Δ40 p53* *in p53 wild-type cases (n=33)*				
<50th percentile	19.6 (13.4–25.9)		33.6 (25.3–41.9)	
>50th percentile	33.4 (25.2–41.8)	0.029	43.1 (38.2–50.0)	0.139

**Table 4:** Multivariate survival analysis of 121 patients with advanced serous ovarian cancer harbouring mutant *p53*

	**Recurrence-free survival**		**Overall survival**	
**Variable**	**HR (95% CI)**	***P*-value**	**HR (95% CI)**	***P*-value**
Δ133p53				
High *vs* low	0.571 (0.362–0.899)	0.016	0.365 (0.182–0.731)	0.004
				
*Age*				
Per year	1.016 (0.995–1.038)	n.s.	1.046 (1.013–1.079)	0.006
				
*Tumour stage*				
III *vs* IV	2.287 (1.373–3.809)	0.001	1.634 (0.771–3.463)	n.s.
				
*Tumour grade*				
I/II *vs* III	1.205 (0.678–2.140)	n.s.	1.964 (0.745–5.178)	n.s.
				
*Residual disease*				
0 *vs* >0	1.729 (1.085–2.753)	0.021	1.909 (0.986–3.697)	0.055

Abbreviations: CI=confidence interval; HR=hazard ratio.
