# Supplementary material for: Δ133p53 is an independent prognostic marker in p53 mutant advanced serous ovarian cancer
Source: Br J Cancer. 2011 Oct 18;105(10):1593–9. doi: 10.1038/bjc.2011.433 (PMC3242533; doi:10.1038/bjc.2011.433)
Supplement: Supplementary Tables [file bjc2011433x1.doc]

**Supplementary Table 1 Primers and probes for *p53* and *p73* isoforms and for the internal control TBP**

|  | **Forward Primer** | **Reverse Primer** | **Probe** |
| --- | --- | --- | --- |
| *∆40p53* | tgtctttcagacttcctgaaaacaac | acagcatcaaatcatccattgc | tctgtcccccttgccgtccca |
| *∆133p53* | cttgtgccctgactttcaactct | cagttggcaaaacatcttgttgag | tctccttcctcttcctacagtactcccctgc |
| *FLp53* | tggaaactacttcctgaaaacaacg | acagcatcaaatcatccattgc | tctgtcccccttgccgtccca |
| *ΔTAp73* | gcgcctaccatgctgtacgt | agattgaactgggccgtgg | tgaccccgcacggcacctc |
| *TAp73* | cctctggagctctctggaacc | tgggccatgacagatgtagtca | cacctacttcgaccttccccagtcaagc |
| *TBP* | cacgaaccacggcactgatt | ttttcttgctgccagtctggac | tgtgcacaggagccaagagtgaaga |

**Supplementary Table 2** Association of *p53* and *p73* isoform expression with *p53* mutational status

|  | ***p53* wild-type**  **median copy number (range)** | ***p53* mutant**  **median copy number (range)** | ***P*-value*** |
| --- | --- | --- | --- |
| *Δ133p53* | 2166.2 (496.3-41003.7) | 2389.3 (122.5-23130.5) | 0.613 |
| *Δ40p53* | 48192.1 (6114.5-11605.0) | 40413.2 (3945.2-321712.4) | 0.524 |
| *FLp53* | 95145.3 (11281.1-257559.4) | 89746.7 (8279.8-551127.8) | 0.697 |
| *ΔNp73* | 153.2 (4.7-1167.9) | 135.9 (9.1-10928.7) | 0.570 |
| *TAp73* | 3077.3 (120.2-44638.8) | 2477.4 (107.0-25485.0) | 0.359 |

*Mann-Whitney U Test

**Supplementary Table 3** Prognostic relevance of p53 and p73 isoforms in the entire group of 154 patients with advanced serous ovarian cancer, univariate survival analyses

|  | **Recurrence-free survival (RFS)** | | | ***P-*value** | **Overall Survival (OS)** | | |  |
| --- | --- | --- | --- | --- | --- | --- | --- | --- |
|  | **Recurrence** | |  |  | **Survival Status** | |  |  |
|  | **No**  **(n = 51)*** | **Yes**  **(n = 103)*** | **Mean RFS in months (95% CI)** |  | **Alive**  **(n = 109)*** | **Dead**  **(n = 45)*** | **Mean OS in months (95% CI)** | ***P-*value** |
| Δ40p53 |  |  |  |  |  |  |  |  |
| < 50 percentile | 18 | 59 | 20.4 (17.3-23.5) |  | 52 | 25 | 36.8 (33.1-40.5) |  |
| > 50 percentile | 33 | 43 | 26.5 (22.4-30.7) | 0.065 | 56 | 20 | 37.8 (34.0-41.5) | 0.535 |
| Δ133p53 |  |  |  |  |  |  |  |  |
| < 50 percentile | 20 | 56 | 20.4 (17.2-23.6) |  | 50 | 26 | 35.3 (31.4-39.2) |  |
| > 50 percentile | 30 | 46 | 25.8 (21.9-29.7) | 0.067 | 57 | 19 | 39.5 (36.0-42.0) | 0.143 |
| FLp53 |  |  |  |  |  |  |  |  |
| < 50 percentile | 19 | 58 | 20.4 (17.3-23.6) |  | 51 | 26 | 35.9 (32.0-39.7) |  |
| > 50 percentile | 32 | 45 | 26.2 (22.2-30.2) | 0.068 | 58 | 19 | 38.6 (35.0-42.1) | 0.202 |
| ΔTAp73 |  |  |  |  |  |  |  |  |
| < 50 percentile | 27 | 47 | 24.7 (20.8-28.5) |  | 54 | 20 | 37.9 (34.3-41.5) |  |
| > 50 percentile | 22 | 51 | 21.9 (18.3-25.5) | 0.435 | 50 | 23 | 37.1 (33.2-41.0) | 0.680 |
| TAp73 |  |  |  |  |  |  |  |  |
| < 50 percentile | 31 | 46 | 25.6 (21.7-29.6) |  | 57 | 20 | 38.2 (34.7-41.7) |  |
| > 50 percentile | 20 | 57 | 20.4 (17.4-23.4) | 0.155 | 52 | 25 | 36.5 (32.7-40.4) | 0.475 |

*Due to missing values, numbers do not add up to a total of 154.

**Supplementary Table 4 Spearman correlation between the expression of N-terminally truncated *p53* and *p73* isoforms in the examined advanced ovarian cancer cases stratified according to *p53* mutational status**

|  | ***p53* mutant cases** | | | | ***p53* wild-type cases** | | | | **Entire group** | | |
| --- | --- | --- | --- | --- | --- | --- | --- | --- | --- | --- | --- |
|  | ***FLp53*** | ***Δ40p53*** | | ***TAp73*** | ***FLp53*** | ***Δ40p53*** | | ***TAp73*** | ***FLp53*** | ***Δ40p53*** | ***TAp73*** |
| *Δ40p53*  ρ*  *P* | 0.964  <0.001 |  | n.s. | | 0.960  <0.001 |  | n.s. | | 0.963  <0.001 |  | n.s. |
| *Δ133p53*  ρ  *P* | 0.322  <0.001 | 0.294  <0.001 | n.s. | | n.s. | n.s. | n.s. | | 0.351  <0.001 | 0.368  <0.001 | n.s. |
| *ΔTAp73*  ρ  *P* | n.s. | n.s. | 0.869  <0.001 | | n.s. | n.s. | 0.929  <0.001 | | n.s. | n.s. | 0.845  <0.001 |

*ρ = Spearman Correlation coefficient
